# Combining E-Nose and Lateral Flow Immunoassays (LFIAs) for Rapid Occurrence/Co-Occurrence Aflatoxin and Fumonisin Detection in Maize

**DOI:** 10.3390/toxins10100416

**Published:** 2018-10-16

**Authors:** Matteo Ottoboni, Luciano Pinotti, Marco Tretola, Carlotta Giromini, Eleonora Fusi, Raffaella Rebucci, Maria Grillo, Luca Tassoni, Silvia Foresta, Silvia Gastaldello, Valentina Furlan, Claudio Maran, Vittorio Dell’Orto, Federica Cheli

**Affiliations:** 1 Department of Health, Animal Science and Food Safety, Università degli Studi di Milano, Via Trentacoste, 2, 20134 Milan, Italy; matteo.ottoboni@unimi.it (M.O.); marco.tretola@unimi.it (M.T.); carlotta.giromini@unimi.it (C.G.); eleonora.fusi@unimi.it (E.F.); raffaella.rebucci@unimi.it (R.R.) vittorio.dellorto@unimi.it (V.D.); federica.cheli@unimi.it (F.C.); 2 ATPr&d S.r.l., Via Ca’ Marzare, 3, Camisano Vicentino, 36043 Vicenza, Italy; mariagrillo@atprd.it (M.G.); lucatassoni@atprd.it (L.T.); silvia.foresta@live.it (S.F.); silviagastaldello@atprd.it (S.G.); claudiomaran@atprd.it (C.M.); 3 CerealDocks S.p.A., Via dell’Agricoltura, 10, Summaga di Portogruaro, 30026 Venezia, Italy; valentinafurlan@cerealdocks.it

**Keywords:** *Zea mays*, aflatoxin, fumonisin, volatile organic compounds (VOCs), electronic nose, co-contamination, discriminant analysis

## Abstract

The aim of this study was to evaluate the potential use of an e-nose in combination with lateral flow immunoassays for rapid aflatoxin and fumonisin occurrence/co-occurrence detection in maize samples. For this purpose, 161 samples of corn have been used. Below the regulatory limits, single-contaminated, and co-contaminated samples were classified according to the detection ranges established for commercial lateral flow immunoassays (LFIAs) for mycotoxin determination. Correspondence between methods was evaluated by discriminant function analysis (DFA) procedures using IBM SPSS Statistics 22. Stepwise variable selection was done to select the e-nose sensors for classifying samples by DFA. The overall leave-out-one cross-validated percentage of samples correctly classified by the eight-variate DFA model for aflatoxin was 81%. The overall leave-out-one cross-validated percentage of samples correctly classified by the seven-variate DFA model for fumonisin was 85%. The overall leave-out-one cross-validated percentage of samples correctly classified by the nine-variate DFA model for the three classes of contamination (below the regulatory limits, single-contaminated, co-contaminated) was 65%. Therefore, even though an exhaustive evaluation will require a larger dataset to perform a validation procedure, an electronic nose (e-nose) seems to be a promising rapid/screening method to detect contamination by aflatoxin, fumonisin, or both in maize kernel stocks.

## 1. Introduction

Maize (*Zea mays* L.) is one of the most important food and feed commodities among cereal crops. However, its diffusion, popularity, and, most of all, safety for consumption are threatened by mycotoxin contamination [1]. Maize is often infected by mycotoxigenic fungi, most notably *Aspergillus*, *Penicillium*, and *Fusarium* spp. [2]. Since it is impossible to fully eliminate the presence of these undesirable substances and the risks linked to them, maximum acceptable concentrations should be set at strict but reasonable-to-achieve levels. Mycotoxin regulations have been established in more than 100 countries around the world [3,4], and the maximum acceptable limits vary greatly from country to country. The European Union (EU) has harmonized regulations among its member states for maximum and/or recommended levels of mycotoxins in both food and feed [5,6]. The purpose is to keep contaminated grain out of the feed and food chains, and to reduce the potential risk associated with mycotoxins. However, more than 300 compounds have been recognized as mycotoxins, of which around 30 are considered a threat to human or animal health. Furthermore, global surveys indicate that more than 70% of the samples of feed and raw feed materials are positive for at least one mycotoxin, emphasizing co-contamination as a big issue for both food safety and public health [7,8,9]. The co-occurrence of mycotoxins in food and feed is explained by three reasons: (i) Most fungi are able to simultaneously produce several mycotoxins; (ii) commodities can be contaminated by several fungi simultaneously or in quick succession; and (iii) the complete diet comprises different commodities [8,10,11].

Among different mycotoxins, aflatoxin (AF) and fumonisin (FM) contamination are quite common in different world areas [10]. AFs are most often detected in Southern Europe, Africa, South Asia and Southeast Asia (average values of positive samples higher than 30%). The highest incidence of fumonisin contamination (more than 50% of positive samples) was found in South America, Southern Europe, Africa, North, and South and Southeast Asia. Although these mycotoxins are quite ubiquitously present in feed materials, the levels of detected mycotoxins are generally low. Multi-mycotoxin contamination, however, is a topic of great concern, as co-contaminated samples might still exert adverse effects on animals due to additive/synergistic interactions of the mycotoxins. The complexity of mycotoxin interactions varies according to the animal species, the level and type of mycotoxin contamination and the length of exposure [10].

This scenario underlines the importance of multi-mycotoxin analysis methods that can also be rapid and user-friendly. Rapid methods for the determination of mycotoxins in cereals are hence highly needed in order to prevent the entry of mycotoxins into food and feed chains. In this respect, the contribution of commercial enzyme-linked immunosorbent assays (ELISA) and lateral flow immunoassays (LFIAs) for mycotoxin determination is important and has been quite well defined [12]. Implementing these assays and combining them with other methods are also essential and necessary. Electronic noses (e-nose) may represent a promising analytical tool for industry, also in combination with immunoassays. An e-nose consists of an array of nonspecific chemical detectors that detect different volatile organic compounds (VOCs) and consequently provides a signal that can be used as a fingerprint of the specific volatile compounds in a sample [13]. E-nose techniques for the detection of fungal infection are based on identifying specific VOCs associated with the growth of fungi on cereal grains [14]. The use of an e-nose could lead to rapid detection of mold contamination in maize at an early phase. The assumption at the basis of this idea is that the growth and biochemical pattern of mycotoxigenic fungi species cause chemical changes in the composition of VOCs [15,16], and a direct correlation between VOCs and mycotoxin concentration in cereals has been observed [15,17,18].

Starting from these assumptions, the aim of this study was to evaluate the potential use of an e-nose for rapid mycotoxin detection in maize, with a focus on aflatoxin and fumonisin. In the study, an e-nose has been combined with commercial LFIAs by which samples were classified as follows: below the regulatory limits, single-contaminated, and co-contaminated.

## 2. Results

### 2.1. Lateral Flow Immunoassay

**Figure 1:**
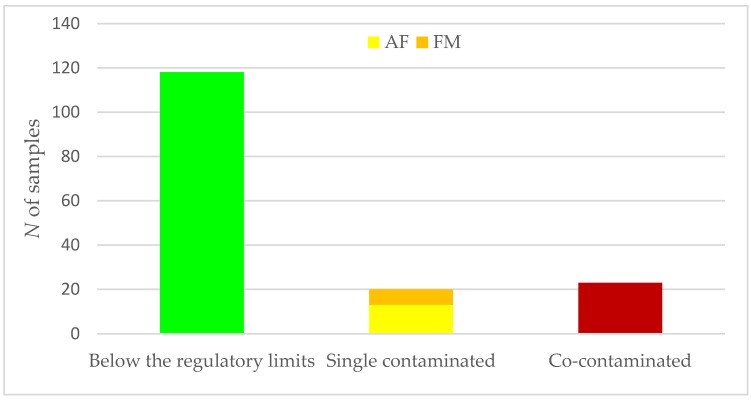
Distribution of maize kernel samples according to the presence of aflatoxin (AF) and fumonisin (FM) determined by LFIA kit (Envirologix™, Portland, ME, USA). Below the regulatory limits (AF < 5 ppb, and FM < 4 ppm); single contaminated, above the regulatory limit for 1 mycotoxin (AF > 5 ppb, or FM > 4 ppm); or co-contaminated, above the regulatory limit for 2 mycotoxins (AF > 5 ppb, and FM > 4 ppm). *N*, number.

The presence of contamination by the two tested mycotoxins, AF and FM, in maize kernels is reported in Figure 1. According to the aim of the study, based on the type and level of mycotoxin contamination, each sample was assigned to one of three classes: below the regulatory limits, single-contaminated, or co-contaminated. Specifically, of the 161 samples analyzed, 118 were below the regulatory limits, 20 were single-contaminated, and 23 were co-contaminated. Among the regular samples (samples below the regulatory limits), the recorded levels for both AF and FM were below the limits for foods established by the EU. Considering single-contaminated samples, thirteen were contaminated by AF, while seven were contaminated by FM.

### 2.2. Electronic Nose

Data from the e-nose were analyzed using DFA, and the performance of the models in predicting maize kernels below the regulatory limits, single contaminated, and co-contaminated with AF and/or FM is summarized in Table 1.

Stepwise variable selection was done to select the e-nose sensors for classifying samples by DFA. The discriminant function used to identify aflatoxin contaminated or below the regulatory limits samples included several e-nose sensors: W1C aromatic, W5S broad-range, W3C Aromatic, W1S Broad-methane, W1W Sulphur-organic, W2S broad-alcohol, W2W Sulph-clor, W3S methane-aliph.

The overall leave-out-one cross-validated percentage of samples correctly classified by the eight variate DFA models for AF was 84.1%. In the case of below the regulatory limits samples, the percentage of samples correctly classified was 89.2%, while in the case of contaminated samples, it was 68.9%.

The discriminant function used to identify FM contaminated or below the regulatory limits samples included seven e-nose sensors, W1C aromatic, W5S broad-range, W5C Arom-aliph, W1W Sulphur-organic, W2S broad-alcohol, W2W Sulph-clor, W3S methane-aliph. The overall leave-out-one cross-validated percentage of samples correctly classified by the seven-variate DFA model for fumonisin was 85.4%. In the case of below the regulatory limits samples, the percentage of samples correctly classified was 87.2%, while in the case of contaminated samples, it was 82.0%.

**Figure 2:**
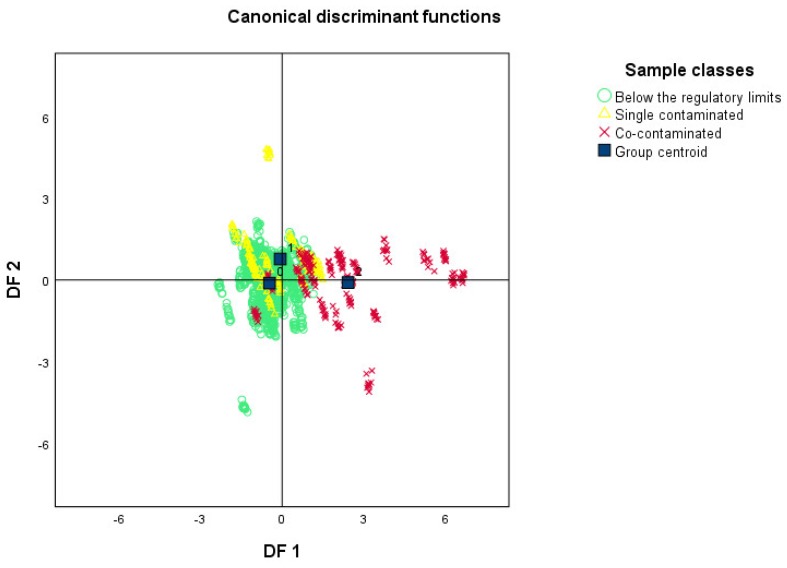
Discriminant analysis scatterplot relative to classification of: 0 = below the regulatory limits (AF < 5 ppb, FM < 4 ppm), green circles (O); 1 = single contaminated, above the regulatory limit for 1 mycotoxin (AF > 5 ppb, or FM > 4 ppm), yellow triangles (Δ); 2 = co-contaminated, above the regulatory limit for 2 mycotoxins (AF > 5 ppb, and FM > 4 ppm), red crosses (×). DF, discriminant function.

A further step in the study was to test the potential of the e-nose in detecting co-contaminated samples. For this purpose, the discriminant function used to identify below the regulatory limits, single-contaminated, and co-contaminated samples included almost all the e-nose sensors (nine of ten), namely: W1C aromatic, W5S broad-range, W3C aromatic, W6S Hydrogen, W1S broad-methane, W1W Sulphur-organic, W2S Broad-alcohol, W2W Sulph-clor, and W3S Methane-aliph. The overall leave-out-one cross-validated percentage of samples correctly classified, by the nine-variate DFA model for the three classes of contamination (below the regulatory limits, single-contaminated, co-contaminated), was 65.2%. In the case of regular samples (below the regulatory limits samples), the percentage correctly classified was 65.4%, while it dropped to 61.0% and 67.4% for single-contaminated and co-contaminated samples, respectively. The same results are shown in the discriminant analysis scatterplot reported in Figure 2. In general, co-contaminated samples (red crosses) tended to group together, confirming results obtained for cross-validation, which, for this class of samples, reached 67% of samples correctly classified. By contrast, regular and single-contaminated samples, shown in the same scatterplot as green circles and yellow triangles, respectively, were more dispersed, indicating higher variability in the VOCs’ profile.

## 3. Discussion

The discriminant function used to identify AF contaminated or below the regulatory limits samples included eight e-nose sensors (W1C aromatic, W5S broad-range, W2S broad-alcohol, and W3S methane-aliph, etc.), that indicated a quite complicated odor profile. It is known that e-nose sensors are nonspecific but can be highly sensitive, responding to a range of different compounds. The conductivity of the polymer changes when molecules are absorbed at the sensor surface. The sensors respond strongly to the presence of alcohols, ketones, fatty acids, and esters, but have a reduced response to fully oxidized materials such as CO_2_, NO_2_, and H_2_O. Thus, the present results are in line with other studies reporting that the production of mycotoxins by particular mold strains is generally associated with the production of volatile substances such as alcohols, aldehydes, ketones, and esters [17]. Moreover, as previously reported by Cheli and co-workers [1], the present work confirms the significant contribution of e-nose metal-oxide-semiconductor (MOS) sensors that are able to detect nitrogen oxides and ozone, which were related to the AF contamination in maize samples quantified by ELISA.

With regard to the eight-variate DFA model for AF, figures obtained in the present study, when compared with the literature [1], seem to indicate that the e-nose in the present study was less effective at distinguishing between contaminated and below the regulatory limits samples. However, in making the comparison, it must be kept in mind that Cheli and co-workers [1] used highly contaminated samples. In that preliminary study, the range of AF contamination was 6–100 ppb, with more than 50% of the samples having >20 ppb of AF.

Moving to the evaluation of the potential use of e-noses for rapid FM detection in maize, the present study indicates that the percentage of samples correctly classified by the seven-variate DFA model for FM was 71%. Although only a few studies have been conducted in this field [15], Keshri and Magan [19] reported that, using an e-nose system, it is possible to differentiate between mycotoxigenic and non-mycotoxigenic strains of such spoilage fungi (e.g.,* Fusarium* spp.) based on their volatile production patterns. However, some of the mycotoxigenic strains used in that study were also able to produce toxins, other than FM (i.e., zearalenone and trichothecene). Similarly, Gobbi and co-workers [20] reported that the e-nose could correctly recognize high FM content (>1000 ppm) and low FM content (<1.6 ppm) in maize cultures (contamination obtained by in vitro incubation of coarsely cracked maize kernels with fumonisin-producing fungi) and provide a fair quantitative estimation. However, in these studies [20], the fungal contamination/spoilage was obtained in vitro from maize cultures inoculated with selected *Fusarium* strains, and sterilized maize cultures were used as reference material/control. By contrast, samples used in the present study were collected from different stockpiles in Italy, which implies that both the fungal strains and the level of contamination were not standardized. Accordingly, in the present work, a higher variability in the odor profile compared to the samples experimentally contaminated in previous studies [20] could be assumed.

Combining the results obtained from the overall leave-out-one cross-validation, it can be suggested that the predictive accuracy of the model was limited. In fact, although below the co-contaminated samples tended to group together, reaching 67% of samples correctly classified, both single-contaminated and regular (below the regulatory limit) samples were more dispersed, indicating a higher variability of the VOCs’ profile. For these classes, the percentages of samples correctly classified were 65% and 61% for regular (below the regulatory limits) and single-contaminated samples, respectively. These figures indicate that more than one third of the samples were wrongly classified, making the method still unsuitable for the purpose of mycotoxin detection. For comparison, Olsson et al. [15] investigated the possibility of using fungal volatile metabolites as indicators of two mycotoxins (ochratoxin A and deoxynivalenol) in barley, using both e-noses and gas chromatography combined with mass spectrometry (GC-MS). In that study, the authors reported that the e-nose misclassified less than 20% of samples (seven of 37 samples) in the case of ochratoxin A, while the deoxynivalenol level could be estimated using a partial least square (PLS) model constructed using the sensor signals from the e-nose. However, in this case, even if several samples were contaminated by both ochratoxin A and deoxynivalenol, detection of co-contamination by ochratoxin A and deoxynivalenol, was not the aim of the study.

Therefore, the present findings seem to indicate that complex chemical patterns of volatile components prevented a complete characterization of the different volatile organic compounds present in regular, single-contaminated and co-contaminated maize, limiting their correct classification to a range values of 60–67%.

## 4. Conclusions

Combining the present results, it can be concluded that e-noses have some potential for detection of selected mycotoxins like aflatoxin and fumonisin in maize. Surprisingly, the e-nose was more effective in detecting co-contaminated samples, reaching 67% of samples correctly classified, while the same value drops to 65% and 61% for regular and single-contaminated samples, respectively.

In the cereal industry, detection of mycotoxin contamination and co-contamination represents a major analytical challenge, and systematic, economical, straightforward cereal tests for rapid and accurate diagnosis of maize safety are needed. At the industrial level, the main question is the choice of the best analytical method for practically enabling rapid decision-making regarding the acceptance or rejection of lots of cereal and ensuring safety standards. Although, for an exhaustive evaluation of e-nose potential for mycotoxins detection other larger datasets are needed, the e-nose seems to be a promising rapid/screening method to detect mycotoxin contamination in maize kernel stocks. Its potential seems improved by its combination with commercially available rapid kit assays for mycotoxin detection. Both are characterized by limited sample preparation, real-time analysis, rapid detection of mold contamination at an early phase, evaluation of co-contamination, easy automation to create models for use as quality control tools, and possible integration into production processes.

## 5. Materials and Methods

### 5.1. Samples Collection and Analysis

For this study, 161 samples of maize (*Zea mays* L.), collected from different stockpiles in Italy between October 2016 and November 2017, were used. Representative samples were obtained according to European Commission Regulation no. 152/2009 [21]. From each sample, two aliquots were subsampled randomly and analyzed with two rapid/screening methods for detection of total aflatoxin and fumonisin contamination or co-contamination. The methods used were: (i) LFIA; and (ii) e-nose.

### 5.2. Lateral Flow Immuno Assay

The first aliquot of each maize sample was immediately analyzed for determination of total aflatoxin (AF) and fumonisin (FM) by commercial LFIA kit (Envirologix™), made up of a lateral flow strip with antibody immobilized on a test zone, the same principle as ELISA tests [12]. Analyses were performed immediately after receiving samples from stockpiles. According to the protocol, quantification of AF and FM was performed in parallel on two representative sub-aliquots. Briefly, each maize sample was finely ground and weighed. After that, according to the protocol for both aflatoxin and fumonisin detection, maize samples were rehydrated in distilled water, and the supernatant was extracted and an aliquot was mixed in a buffer and incubated for 2 min. Finally, a colorimetric strip was added for 4 min or 5 min (for aflatoxin or fumonisin, respectively) and then read by QuickScan Envirologix^TM^. The detailed protocol is reported in Table 2. For both AF and FM detection, the base range protocol was adopted (AF detection from 2.7 ppb to 30 ppb; FM detection from 1.5 ppm to 7 ppm). Each sample was analyzed in duplicate. Results obtained for AF and FM were used in classifying each sample as follows: (i) Samples below the regulatory limits; (ii) single-contaminated; or (iii) co-contaminated samples. Further details about the grouping (meet regulatory limit, above regulatory limit for 1 mycotoxin, and above regulatory limit for 2 mycotoxins) of the different samples according to the LFIA results are reported in the statistical analysis section.

### 5.3. Electronic Nose Analysis

The second aliquots of maize samples were stored at −18 °C in vacuum-sealed conditions prior to e-nose analysis, in order to prevent the development of further odors and off-odors that could affect the reliability of the results. For the e-nose analysis, 10 g of each sample of maize kernels was placed into an airtight 20 mL glass vial and sealed with a chlorobutyl/PTFE magnetic cap. The e-nose’s pipe was connected to the vials with a needle: Between the needle and the pipe, a filter was inserted in order to block the dust that was present in some samples and could ruin the sensors. One more needle was inserted into each vial in order to avoid creating negative pressure inside the vial.

The analysis was performed on a portable electronic nose 3 (PEN 3) model e-nose from Airsense Analytics GmbH (Schwerin, Germany). The sensor array consisted of 10 metal-oxide-semiconductor (MOS) chemical sensors made of a ceramic substrate heated by a wire resistor and coated with a metal oxide semiconducting film. At operating conditions, interactions between the volatiles from the head space and the sensor’s surface induced changes in the conductance of the semiconductor. Thus, the ratio G/G0 (in which G and G0 represent the resistance of a sensor when detecting a gas and when inhaling clean air, respectively) was recorded by the e-nose dedicated software. The characteristics of the e-nose sensor array are listed in Table 3. Preliminary trials were performed to evaluate the sensitivity of e-nose sensors using experimentally contaminated maize kernels [1].

After an equilibration period (2 h to 3 h) at room temperature, the gas in the head space of each vial was analyzed by the e-nose with a flow of 400 mL/min. During the analysis (60 s), each sensor’s signal was recorded when the sensor’s response curve showed a stabilized conductance for at least 10 s. During this time (measurement time), data from the raw sensor signals for each maize sample were recorded (with a 1 s interval). Thus, each sample was analyzed in duplicate and run three times (i.e., 6 observation per samples). The model has been fitted with the mean of 6 observations for each second (N of measurements) per sample (i.e., the sample odor profile).

Ten different descriptors, representing each sensor of the e-nose, were used to detect mycotoxin contamination. After acquisition, all sensor signal measurements were collected in Excel files and used for dataset assembly.

### 5.4. Statistical Analysis

Based on the levels of AF and FM measured by LFIA, each sample was assigned to one of three classes: (i) below the regulatory limits (AF < 5 ppb, and FM < 4 ppm); (ii) single-contaminated, above the regulatory limit for 1 mycotoxin (AF > 5 ppb, or FM > 4 ppm); or (iii) co-contaminated, above the regulatory limit for 2 mycotoxins (AF > 5ppb, and FM > 4 ppm).

Discriminant function analysis (DFA) was done on the e-nose data. The analysis was done on the original dataset with 9660 measurements (161 samples × 10 MOS × 2 replicates × 3 runs). All analysis was done with IBM SPSS 22.0 (IBM Corp., Armonk, NY, USA) predictive analytics software. In DFA, the aim is basically to build up a predictive model for group membership. The model is composed of a discriminant function based on linear combinations of predictor variables. In addition, those predictor variables provide the best discrimination between groups. The stepwise variable selection was conducted to ensure getting the most significant variables to carry out DFA. This method shows the variables that give the best results for DFA, by regarding the Wilk’s test results. Leave-out-one-sample cross-validation was also done.

## Figures and Tables

**Table 1:** Discriminant function analysis classification performances.

Group	Measurements, *N*	*N* (% of Sample Correctly Classify)		
**Aflatoxins**		**Below the regulatory limit (<5 ppb)**	**Above the regulatory limit (>5 ppm)**	
Below regulatory limit (<5 ppb)	1070	954 (89.2)	116 (10.8)	
Above regulatory limit (>5 ppb)	360	112 (31.1)	248 (68.9)	
**Fumonisins**		**Below the regulatory limit (<4 ppm)**	**Above the regulatory limit (>4 ppm)**	
Below regulatory limit (<4 ppm)	540	471 (87.2)	69 (12.8)	
Above regulatory limit (>4 ppm)	300	54 (18.0)	246 (82.0)	
**Class assigned**		**Below the regulatory limit**	**Above the regulatory limit for 1 mycotoxin**	**Above the regulatory limit for 2 mycotoxin**
Below regulatory limit	1180	772 (65.4)	403 (34.2)	5 (0.4)
Above regulatory limit for 1 mycotoxin	200	64 (32.0)	122 (61.0)	14 (7.0)
Above regulatory limit for 2 mycotoxin	230	26 (11.3)	49 (21.3)	155 (67.4)

*N*, number.

**Table 2:** Aflatoxin and fumonisin detection in maize by lateral flow immunoassays (LFIA): base range protocol.

Mycotoxin Detected	Weight and Dilution	Fully Wet Sample, then Mix	Clarify	Protocol	Pre-Mix as Noted, then Transfer 200 µL to Reaction Tube	Pre-Mix Sample in Blue Dilution Tube Followed by Transfer to Clear Reaction Tube	Add Reaction Tube to Incubator Set at 22 °C	Add Strip for	Read Results in
AF	50 g sample in EB17 pouches with 150 mL water. Immediately shake vigorously for 10 s by hand	1 min highest speed on shaker table	Centrifuge	Base Range 0–30 ppb	Pre-Mix 100 µL DB5 buffer + 100 µL extract in Reaction Tube	Pre-Mix 2.5 mL buffer + 50 µL extract. Transfer 200 µL	Acclimate tube for 2 min	4 min	QuickScan Envirologix^TM^
FM	50 g sample in 250 mL water	1 min highest speed on shaker table	Settle	Base Range 1.5 ppm to 7.0 ppm	Pre-Mix 100 µL DB5 buffer + 100 µL extract in Reaction Tube	Pre-Mix 2.5 mL buffer + 50 µL extract. Transfer 200 µL	Acclimate tube for 2 min	5 min	QuickScan Envirologix^TM^

**Table 3:** Metal-oxide-semiconductor (MOS) sensor array of portable electronic nose 3 (PEN 3).

Sensor Number in Array	Sensor Name	Description	Reference
W1C	Aromatic	Aromatic compound	Toluene 10 ppm
W5S	Broadrange	Broad range sensitivity reacts to nitrogen oxides and ozone, very sensitive with negative signal	NO_2_ 10 ppm
W3C	Aromatic	Ammonia, used as sensor for aromatic compounds	Benzene 10 ppm
W6S	Hydrogen	Mainly hydrogen, selective (breath gases)	H_2_ 100 ppm
W5C	Arom-aliph	Alkanes, aromatic compounds, less polar compounds	Propane 1 ppm
W1S	Broad-methane	Sensitive to methane (environment) 10 ppm. Broad range	CH_4 _100 ppm
W1W	Sulphur-organic	Reacts on sulphur compounds. Sensitive to many terpenes and sulphur organic compounds, which are important for smell, limonene, pyrazine	H_2_S 0.1 ppm
W2S	Broad-alcohol	Detects alcohol, partially aromatic compounds, broad range	CO 100 ppm
W2W	Sulph-clor	Aromatic compounds, sulphur organic compounds	H_2_S 1 ppm
W3S	Methane-aliph	Reacts on high concentration >100 ppm, sometimes very selective (methane)	CH_4_ 10 ppm
